# The Role of Bystander Cardiopulmonary Resuscitation: A Meta-Analysis

**DOI:** 10.1155/emmi/5591055

**Published:** 2025-07-26

**Authors:** Xi Chen, Zexi Zou, Xueyi Wen, Linfei Li, Yuanyuan Liang

**Affiliations:** ^1^Department of Emergency Medicine, Affiliated Hospital of Zunyi Medical University, Zunyi, Guizhou, China; ^2^School of Nursing, Zunyi Medical University, Zunyi, Guizhou, China

**Keywords:** bystander cardiopulmonary resuscitation, out-of-hospital cardiac arrest, return of spontaneous circulation, survival

## Abstract

**Objective:** This meta-analysis systematically evaluated the impact of bystander cardiopulmonary resuscitation (BCPR) on the survival of patients with out-of-hospital cardiac arrest (OHCA) and related factors.

**Methods:** A computerized search of China National Knowledge Infrastructure (CNKI), Wanfang Database, Chinese Biomedical Literature Database (CBM), PubMed, and Embase was conducted from the database's inception to May 1, 2023. The study included observational studies of patients who experienced OHCA and were successfully resuscitated using BCPR, following the predetermined criteria for inclusion and exclusion. The quality of the included studies was assessed using the Newcastle–Ottawa scale, with odds ratios (ORs) and 95% confidence intervals (95%CI) used as effect size measures. The data were statistically analyzed using Review Manager 5.4 software.

**Results:** Fourteen observational studies were included in this study, involving 253,247 cases of OHCA. The primary outcome measure was survival to discharge or survival at 30 days. A meta-analysis was conducted to analyze the data from these 14 studies. The findings indicated that the no-BCPR group had a significantly reduced survival rate compared to the BCPR group (OR: 1.72, 95% CI: 1.40–2.12, *p* < 0.05). Secondly, the study examined 14 studies that focused on prehospital return of spontaneous circulation and neurological recovery before they reached the hospital. The findings revealed that patients who received BCPR had a higher rate of prehospital ROSC (OR: 2.06, 95% CI: 1.66–2.57, *p* < 0.05) and experienced better neurological recovery (OR: 2.03, 95% CI: 1.67–2.47, *p* < 0.05) compared to those who did not receive BCPR. This difference was found to be statistically significant.

**Conclusion:** BCPR can potentially enhance the likelihood of survival for patients experiencing OHCA. BCPR can offer patients an opportunity for both survival and favorable neurological recovery during the time when emergency medical services (EMS) respond. Given the existing circumstances, it is advisable to enhance the promotion and training of public CPR and improve the prevalence of bystander CPR in society since this is expected to yield substantial social advantages.

## 1. Introduction

Out-of-hospital cardiac arrest (OHCA) is a worldwide health issue, with survival rates ranging from 2% to 22% across different nations or regions. The mortality rate from OHCA is expected to increase due to the aging population globally [1]. OHCA can be defined as the cessation of effective mechanical activity of the heart, resulting in the absence of blood circulation, and it happens outside a medical facility. The American Heart Association reported that over 356,000 occurrences of OHCA registered each year in the United States. There are more than 70,000 cases in Japan annually [2], while Europe sees 275,000 cases annually. However, only 29,000 of these European cases survived and were discharged from the hospital [3]. In 2014, emergency medical services (EMS) in England reported treating 28,729 OHCA occurrences, corresponding to a rate of 53 cases per 100,000 residents. Unfortunately, only 7.9% of these victims survived and were discharged from the hospital [4]. The global incidence of OHCA treated by EMS is approximately 34.4% in Europe, 53.1% in North America, 59.4% in Asia, and 49.7% in Australia. The survival rates for these regions are 7.6% in Europe, 6.8% in North America, 3.0% in Asia, and 9.7% in Australia [5].

Although there have been significant advancements in emergency medicine and resuscitation techniques, the chances of surviving an OHCA are still quite low. The outcomes following OHCA are greatly influenced by the timing of intervention, emphasizing the crucial need for early action. The chain of survival strategy for treating OHCA focuses on a comprehensive approach to care. This approach highlights the importance of immediate interventions such as CPR and defibrillation, initiated by witnesses before medical professionals arrive. These interventions significantly impact on improving the patient's prognosis and are considered one of the most crucial factors that can be modified to enhance OHCA survival rates [6]. The primary objective of cardiopulmonary resuscitation (CPR) is to produce sufficient blood circulation to essential organs, including the brain and heart, until spontaneous circulation can be reinstated [7]. Early defibrillation has been shown to significantly increase survival rates, reduce the risk of brain injury, and potentially extend the window of opportunity for successful defibrillation [8].

Optimal resuscitation is most successful within the initial 20 min. However, in the present national healthcare setting, individuals experiencing cardiac arrest outside of a medical facility usually only receive chest compressions upon the arrival of an ambulance, potentially resulting in a missed opportunity for effective resuscitation during this interval. The median EMS response time is a minimum of 5 min. If the patient is not treated prior to the arrival of EMS, the heart, brain, and other essential organs are deprived of blood supply for an extended duration. This often poses challenges to the patient's recovery from autonomous circulation. The US National Academy of Medicine and the International Liaison Committee on Resuscitation (ILCOR) stress the need to increase bystander cardiopulmonary resuscitation (BCPR) as a crucial global objective [9].

This study utilized meta-analysis to examine the impact of BCPR on the survival outcomes of individuals experiencing cardiac arrest outside of a medical facility. The study examines the impact of BCPR on the recovery of patients' prehospital autonomic respiratory circulation. It emphasizes the significance of BCPR in enhancing the survival rate of patients with OHCA. The findings have important implications for both social and clinical efforts to improve survival rates in such cases. This study enhances community awareness of the significance of BCPR. It increases the likelihood of BCPR when faced with a cardiac arrest patient outside of a medical facility.

## 2. Methods

### 2.1. Data Sources

We conducted a comprehensive search across many Chinese and English databases to identify observational studies that investigated the impact of BCPR on the survival rates of patients experiencing OHCA.

### 2.2. Search Strategy

We conducted computer searches on various data platforms including Pubmed, Embase, CNKI, Wanfang Data Knowledge Service Platform, and CBM. The searches were performed from the inception of each repository until May 2023. Additionally, we expanded the sample size by reviewing the included literature and relevant references. Key search terms in English include out-of-hospital cardiac arrest, out-of-hospital ventricular fibrillation, and bystander cardiopulmonary resuscitation. The retrieval is conducted using a combination of subject terms and free words. Taking the PubMed retrieval strategy as an example, please refer to Table 1.

### 2.3. Eligibility Criteria

#### 2.3.1. Incorporate Standards

(1) Study design: observational (including cohort studies or case–control studies); (2) patient participants: Patients who experience sudden cardiac arrest outside of a medical facility (aged 18 or older, nontraumatic cardiac arrest, nontoxic-induced cardiac arrest); (3) intervention: The study included a test group consisting of OHCA patients who got CPR from a witness, and a control group consisting of patients who did not get CPR from a witness; (4) outcome indicator: Survival discharge rate (or 30-day survival rate).

#### 2.3.2. Exclusion Criteria

(1) Individuals lacking the outcome above indications; (2) unrecorded literature; (3) reviews, children's studies, animal studies, replicated literature, case reports, and ongoing research.

### 2.4. Data Extraction

Two researchers conducted literature screening separately, strictly following the criteria for including and excluding literature. Any differences in their findings were resolved through discussion with a third researcher. If there were any gaps or missing information in the study, we contacted the target article's author via contact or email to get as much relevant data as possible. The included extracts encompass the following details: the primary author, the study's kind, the year of publication, the nation or location, essential information about the sample such as age, EMS response time, number of survivors, etc., the intervention employed, and the outcome indicators.

### 2.5. Quality Assessment

The quality of the 14 publications was assessed using a modified version of the Newcastle–Ottawa Quality Assessment Scale for Cohort Studies [10], which comprises eight criteria divided into three categories. The total score was 9. The section on study population selection includes four measures (4 points), the section on between-group comparability includes one measure (2 points), and the section on outcome/exposure factors includes three measures (3 points). A study with a score of ≥ 6 is considered high quality, with higher scores indicating a lower risk of bias and higher quality of the literature. For this study, two researchers assessed the literature's quality and categorized the risk of bias for each evaluation item as “low risk,” “medium risk,” or “high risk.” If there were any disagreements between the two researchers during the evaluation, they would consult with a third researcher to decide.

### 2.6. Statistical Analysis

The data were evaluated using Review Manager 5.4 software. The results were presented as odds ratios (ORs) and their corresponding 95% confidence intervals (95%CIs). The I-square (*I*^2^) test was employed to ascertain the presence of statistical heterogeneity in the included literature. If the *I*^2^ value exceeded 50% and *p* was less than 0.05, it signified the presence of statistical heterogeneity in the literature that was considered. In such cases, a meta-analysis was conducted using a random-effects model. A fixed-effects model was employed for the meta-analysis if the *I*^2^ value was equal to or less than 50%, and a *p* value greater than 0.05 showed the absence of statistical heterogeneity in the literature included. Sensitivity analysis was employed to assess the stability and reliability of the analysis findings. A significance level of *p* < 0.05 was utilized to determine the statistical significance of the results.

## 3. Results

### 3.1. Literature Search and Selection

**Figure 1:**
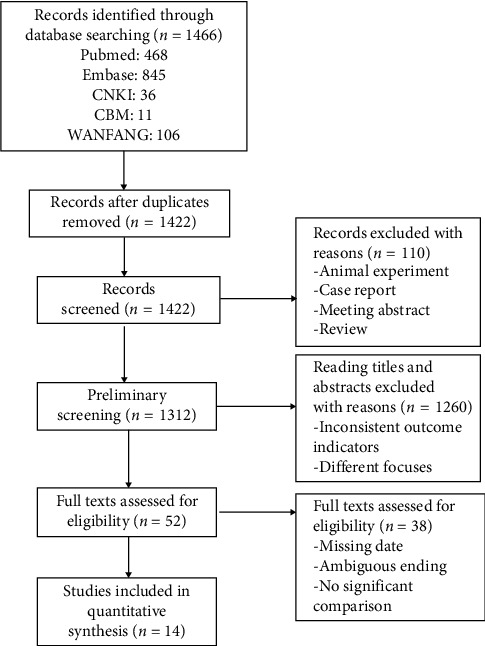
Flow diagram of literature search and selection process of the studies.

After conducting an initial database search, we found 1466 relevant literature titles for our study. These titles included 468 from PubMed, 845 from Embase, 36 from China Knowledge Network, 106 from Wanfang Data Knowledge Service Platform, and 11 from China Biomedical Literature Database. After applying our inclusion and exclusion criteria, we selected 14 documents that met our requirements. These documents contained a total of 253,247 patients. The precise procedure of literature screening is illustrated in Figure 1.

### 3.2. Sample Characteristics

Table 2 displays the fundamental attributes of the 14 research incorporated in the analysis. The studies consist of 2 papers written in Chinese [10, 21], 1 piece in German [16], and 11 publications in English [1, 8, 11–15, 18–20, 22, 23]. These articles were published between 1995 and 2023. The sample sizes in these studies vary from 852 to 93,623. The regions covered in these studies include China, Denmark, the United States, South Korea, Germany, Japan, and Singapore. One of the research studies, conducted by SangHunKim [15], examined the difference in survival rates between individuals who received BCPR with or without an automated external defibrillator (AED). Sungbea Moon's study [19] also examined the impact of EMS response time on the survival of patients with OHCA after receiving BCPR. Toshihiro [20] examined the impact of whether or not the direction of BCPR by the dispatcher affects the survival of patients with OHCA. Seven studies [8, 10, 14, 17–19, 21]recorded the frequency of on-site ROSCs (return of spontaneous circulation) in patients who experienced OHCA. Additionally, all seven studies [14, 17–22]included in the analysis contained comprehensive follow-up data on the neurological recovery (refers to the process by which patients gradually regain normal neurological functions following CPR, including significant improvements in motor skills, sensory functions, cognitive abilities, and daily living capabilities; e.g., cerebral performance category score of 1 or 2) of OHCA patients.

### 3.3. Meta-Analysis Results

#### 3.3.1. Survival of All OHCAs

**Figure 2:**
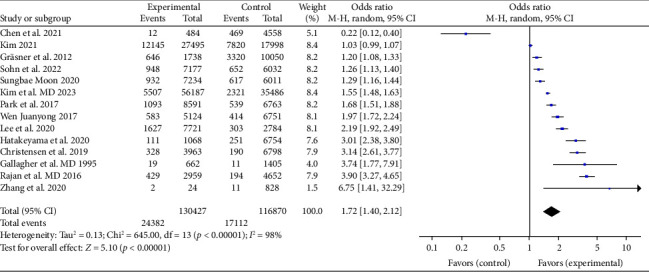
Forest plot of the effect of BCPR on the survival rate of OHCA patients.

All 14 research examined the impact of whether a witness at the scene administered CPR on the survival of patients with OHCA until they were discharged from the hospital or survived for 30 days. The studies showed a significant variation in their statistical results (*I*^2^ = 98%, *p* < 0.001). We conducted a meta-analysis using a random-effects model. The findings indicate that the survival rate was significantly higher in the group that received BCPR compared to the group with no-BCPR (OR: 1.72,95% CI: 1.40–2.12, *p* < 0.05). This difference is statistically significant, as depicted in Figure 2.

#### 3.3.2. Sensitivity Analysis

**Figure 3:**
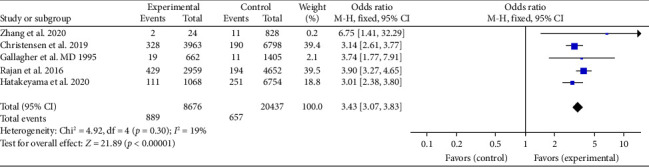
Forest plot of the effect of BCPR on the survival rate of OHCA patients after sensitivity analysis.

A sensitivity analysis was conducted by changing the effect model, and the results were similar to previous findings (*p* < 0.05), indicating robust analysis outcomes. In the sensitivity analysis, nine studies [1, 14–19, 21, 22] significantly impacted heterogeneity, After removing these studies, *I*^2^ dropped to 19%. As shown in Figure 3, the meta-analysis results using a fixed-effects model demonstrated a statistically significant difference in survival rates between BCPR and no-BCPR (OR: 3.43, 95% CI: 3.07–3.83, *p* < 0.05).

#### 3.3.3. ROSC of All OHCAs

**Figure 4:**
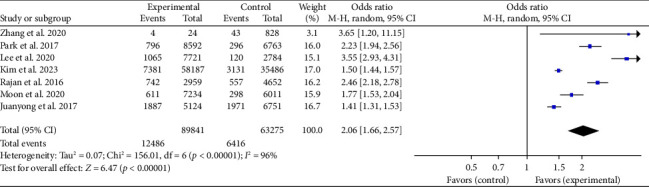
Forest plot of the effect of BCPR on prehospital ROSC of OHCA patients.

Seven studies we included in our analysis recorded the number of patients who achieved an on-site ROSC. To further examine the impact of BCPR, we conducted a meta-analysis focusing on this outcome. Given the significant diversity (*p* < 0.001, *I*^2^ = 96%), we employed a random-effects model. The meta-analysis findings indicate that patients with BCPR had significantly greater odds of achieving spontaneous respiratory circulation at the scene than those with no-BCPR. This difference is supported by the statistical significance (OR: 2.06; 95% CI: 1.66–2.57; *p* < 0.05) shown in Figure 4.

#### 3.3.4. Neurological Recovery of All OHCAs

**Figure 5:**
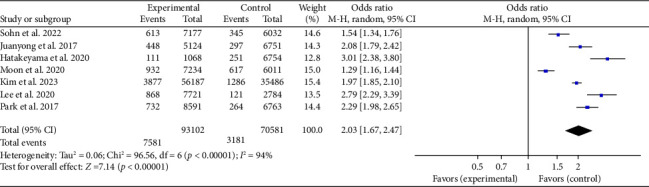
Forest plot of the effect of BCPR on the neurological recovery of OHCA patients.

Seven papers we considered had complete documentation of patients' neurological recovery. Since neurological recovery is a crucial determinant for the survival of patients who have BCPR, as it directly affects the quality of their survival, we conducted a meta-analysis specifically for this purpose. Given the significant heterogeneity (*p* < 0.001, *I*^2^ = 94%), we employed a random-effects model. The meta-analysis results demonstrated a significant statistical difference in the chances of achieving a favorable neurological recovery with BCPR compared with that of no-BCPR (OR: 2.03; 95% CI: 1.67–2.47; *p* < 0.05), as illustrated in Figure 5.

## 4. Discussion

OHCA is widely acknowledged as a significant global public health issue. Despite extensive endeavors in academia and the public sphere, OHCA remains associated with a significant mortality rate in numerous countries. It is recognized as a leading cause of death [19]. This paper examines the effects of BCPR on the survival and recovery rates of OHCA patients. It compares and discusses the impact of BCPR on the number of patients who survive until discharge and those who experience prehospital ROSC. The findings indicate that BCPR performed by bystanders can significantly enhance the recovery of spontaneous breathing and circulation before arrival at the hospital and an increased survival rate until discharge for OHCA patients. The function and impact of BCPR are substantial.

The survival of patients with OHCA was strongly influenced by several factors, as highlighted in an 18-year Swedish study [23]. These factors included short rescue delays, effective CPR performed by bystanders, being female, and the location of the incident:

1. Moon et al. [19] examined the impact of EMS response time on the survival rate of OHCA patients following BCPR. The findings indicate that prompt access to EMS unquestionably enhances patient survival. However, as the time gap between EMS interventions widens, cardiac arrest patients who receive BCPR exhibit significantly higher rates of survival until discharge and favorable neurological recovery outcomes compared with no-BCPR during the EMS intervention period. When witnesses do CPR, the period in which there is a greater chance of improving neurological outcomes and increased chances of survival is considerably longer. This finding aligns with the results of previous studies [8, 14, 24]. Furthermore, the study found a substantial increase in neurological recovery and survival discharge rates increase significantly in the group with BCPR. This indicates that performing CPR immediately after seeing a cardiac arrest leads to improved chances of life and better neurological outcomes in the specific demographic being studied.
2. Kim [15] examined the impact of administering an AED during BCPR on survival rates. The findings indicate that the first utilization of AEDs greatly enhances the survival rate upon discharge for those experiencing OHCA. However, the limited dissemination of AED-related information poses challenges for the general people in effectively administering high-quality CPR with public AEDs. Thus, in the event of cardiac arrest, it is imperative to promptly administer chest compressions to facilitate adequate blood flow prior to the arrival of medical professionals. Additionally, defibrillation may be employed on individuals experiencing ventricular fibrillation to restore normal heart rhythm.
3. Hatakeyama et al. [20] included an analysis of the impact of BCPR on the survival rates of patients with OHCA, specifically examining if the presence of a dispatcher influenced the outcomes. The study conducted in Osaka City utilized a comprehensive registry of OHCAs to show that individuals with BCPR who relied on instructions from a CPR instructor did not have higher survival rates compared with no-CPR. Furthermore, the study suggests that the quality of BCPR for those who require instruction may not be adequate to improve the chances of favorable neurological outcomes. However, this outcome could be attributed to the possibility that witnesses needing guidance from a dispatcher may have less experience than BCPR without instructions.

The significance of the first eyewitness is particularly notable given all of this research. Based on a multicenter prospective observational study conducted in 13 emergency departments across six provinces and cities in different regions of China (northern, southern, eastern, and southwestern) [25], the effectiveness of CPR in patients experiencing emergency cardiac arrest is notably low. However, it was found that BCPR plays a crucial role in enhancing the chances of survival for these patients. The German study [16] revealed that implementing resuscitation techniques like BCPR before the arrival of the ambulance service led to a 4% increase in patient survival rate and an 8% increase in the ROSC rate, compared to interventions that began after the ambulance service had arrived. Since 1995, it has been emphasized that the majority of cardiac arrests happen outside of medical facilities. Consequently, a nonmedical individual or layman [13] will probably be called upon to provide CPR. Therefore, it is crucial to provide CPR training to witnesses. Prior research has indicated that the impact of BCPR on outcomes of OHCA may differ according to factors such as patient age, type of witness, and OHCA characteristics. However, the most crucial factor influencing these results is the quality of BCPR [17]. Although public healthcare services such as EMS, AEDs, and dispatcher guidance are advancing and improving, there is a need to enhance the overall first aid knowledge in the community through various training methods. This will help increase bystanders' ability to quickly recognize emergency patients' conditions during crises, enabling them to respond promptly and administer high-quality CPR. Ultimately, this will improve the community's survival rate of OHCAs.

In existing meta-analyses, Yu et al. [26] demonstrated that community interventions are associated with higher survival rates after OHCA, with enhanced effects observed in regions with healthcare service interventions. Simmons et al. s research [27] revealed that community-based interventions positively influence four key factors: bystander CPR rates, bystander AED usage rates, survival rates, and favorable neurological outcomes post OHCA. Building on these findings, our study uniquely incorporates the analysis of patient ROSC, adding depth to the existing body of knowledge by including a wider range of variables. Specifically, this study focuses on the implementation and outcomes of BCPR in East Asia, providing critical empirical evidence that supports the formulation of more effective public health strategies tailored to this region's unique context. Additionally, this study underscores the importance of public training in improving the survival rates of OHCA patients.

## 5. Limitations

However, several limitations should be acknowledged. (1) Heterogeneity of included studies: The high heterogeneity among the included studies may be attributed to differences in medical emergency systems and the level of public CPR education across various regions. Additionally, variations in witness characteristics (such as age, gender, weight, occupation), CPR quality, the timing of initial response, and total duration of compressions further contribute to the heterogeneity, making it difficult to collect and analyze this information consistently. (2) Location of cardiac arrest: The analysis did not consider the location of cardiac arrest (such as urban/rural ratio or distribution of high-rise buildings), time of occurrence, or patient demographic information (gender and age) to compare survival rates across different groups. (3) Prehospital factors: This study primarily focused on prehospital factors and did not account for in-hospital treatment and subsequent resuscitation efforts (such as target temperature management and extracorporeal membrane oxygenation), which could affect outcomes for OHCA patients. Future research should include more variables, considering the differences in postarrest care capabilities among hospitals in different countries and incorporate in-hospital treatment data to adjust the analysis results accordingly [19]. (4) Dispatcher-assisted CPR: The potential impact of dispatcher-assisted CPR on the survival rates of OHCA patients was not considered. While dispatcher-assisted CPR is an important factor in improving patient outcomes, this analysis did not include its influence. Future studies should consider this variable to provide a more comprehensive understanding of the factors affecting the survival of OHCA patients.

## 6. Conclusions

In conclusion, it is imperative to promote widespread awareness and proficiency in CPR through various means in the country. Additionally, legal provisions should be established to alleviate concerns among individuals capable of performing rescue efforts. These measures will enhance the spirit of solidarity in society and foster excellence and bravery. At the same time, it is important to strengthen prehospital medical personnel's training in utilizing information technology and guidance systems. Public education on defibrillation should also be prioritized. Moreover, the effective use of emergency information platforms and the ongoing improvement of prehospital emergency care are critical to reducing OHCA-related mortality.

## Figures and Tables

**Table 1:** PubMed search strategy.

1. Out-of-hospital ventricular fibrillation
2. Out-of-hospital asystole
3. Out-of-hospital pulseless breathing
4. Out-of-hospital cardiac arrest OR OHCA
5. Bystander cardiopulmonary resuscitation OR BCPR
6. (1 or 2 or 3 or 4) and 5

*Note:* BCPR, bystander cardiopulmonary resuscitation.

Abbreviation: OHCA, out-of-hospital cardiac arrest.

**Table 2:** Characteristics of the studies included in the meta-analysis.

Serial number	First author	Publication year	Country (region)	Control/experimental	Age (median)	EMS response time (min)	Outcome indicator	ROSC on site (control/experimental)	Neurological recovery status (control/experimental)	NOS
1	Zhang et al. [11]	2020	China	828/24	58.9	13.12	ROSC on site, 30-day survival rate	43/4	N/A	7
2	Christensen et al. [12]	2019	Denmark	6798/3963	67	N/A	30-day survival rate, 1-year survival rate	N/A	N/A	6
3	Gallagher et al. [13]	1995	America	1405/662	N/A	N/A	Survival to hospital discharge	N/A	N/A	6
4	Park et al. [14]	2017	Korea	6763/8591	71.2	7	ROSC on site, survival to hospital discharge, neurological recovery	296/796	264/732	8
5	Kim [15]	2021	Korea	17,998/27,495	70	N/A	Survival to hospital discharge	N/A	N/A	6
6	Gräsner et al. [16]	2012	German	10,050/1738	64.9	N/A	Whether ROSC on admission, survival to hospital discharge	N/A	N/A	6
7	Lee et al. [17]	2020	Korea	2784/7721	65.4	N/A	Initial heart rate on site, ROSC on site, survival to hospital discharge, neurological recovery	120/1065	121/868	8
8	Kim et al. [18]	2023	Korea	35,486/58,187	73.5	7	Prehospital ROSC, survival to hospital discharge, neurological recovery	3131/7381	1286/3877	8
9	Rajan et al. [8]	2016	Denmark	4652/2959	67	N/A	ROSC, 30-day survival rate	557/742	N/A	7
10	Moon et al. [19]	2020	Asia (multicenter)	6011/7234	N/A	N/A	Survival to hospital discharge, neurological recovery	298/611	617/932	7
11	Hatakeyama et al. [20]	2020	Japanese	6754/1068	65	N/A	30-day survival rate, neurological recovery	N/A	251/111	7
12	Wen et al. [21]	2017	North America	6751/5124	65.3	5.9	ROSC on site, recovery rate, neurological recovery	1971/1887	297/448	7
13	Sohn et al. [22]	2022	Korea	6032/7177	68	N/A	Survival to hospital discharge	N/A	345/613	7
14	Chen et al. [1]	2021	China	4558/484	78	N/A	Prehospital ROSC, survival to hospital admission, survival to discharge	N/A	N/A	6

*Note:* A multicenter study encompassing Tokyo, Osaka, Honshu Island, Aichi prefecture, Seoul, Taipei, and Singapore.

Abbreviations: EMS, emergency medical services; N/A, not available; ROSC, return of spontaneous circulation.

## Data Availability

The data that support the findings of this study are available from the corresponding author upon reasonable request.
